# Toward the identification of a phytocannabinoid-like compound in the flowers of a South African medicinal plant (*Leonotis leonurus*)

**DOI:** 10.1186/s13104-020-05372-z

**Published:** 2020-11-10

**Authors:** E. Hunter, M. Stander, J. Kossmann, S. Chakraborty, S. Prince, S. Peters, Bianke Loedolff

**Affiliations:** 1 Department of Genetics, Institute of Plant Biotechnology, Faculty of AgriSciences, Stellenbosch University, Stellenbosch, South Africa; 2 Central Analytical Facilities, Stellenbosch University, Stellenbosch, South Africa; 3 Department of Human Biology, Faculty of Health Sciences, University of Cape Town, Cape Town, South Africa

**Keywords:** Adrenoyl-EA, Traditional medicine, Phytochemistry, Flowers, Cannabis, Phytocannabinoids, *Leonotis leonurus*

## Abstract

**Objective:**

Current global trends on natural therapeutics suggest an increasing market interest toward the use and discovery of new plant-derived therapeutic compounds, often referred to as traditional medicine (TM). The Cannabis industry is currently one such focal area receiving attention, owing to the occurrence of phytocannabinoids (pCBs) which have shown promise in health-promotion and disease prevention. However, the occurrence of pCBs in other plant species are often overlooked and rarely studied. *Leonotis leonurus* (L.) R. Br*.* is endemic to South Africa with a rich history of use in TM practices amongst indigenous people and, has been recorded to induce mild psychoactive effects akin to Cannabis. While the leaves have been well-reported to contain therapeutic phytochemicals, little information exists on the flowers. Consequently, as part of a larger research venture, we targeted the flowers of *L. leonurus* for the identification of potential pCB or pCB-like compounds.

**Results:**

Flower extracts were separated and analyzed using high performance thin layer chromatography (HPTLC). A single pCB candidate was isolated from HPTLC plates and, using liquid chromatography coupled to tandem mass spectrometry (LC–MS/MS), we could successfully group this compound as a fatty amide and tentatively identified as 7,10,13,16-Docosatetraenoylethanolamine (adrenoyl-EA), a known bioactive compound.

## Introduction

Increasing interest from developed economies in the use of TMs has created the second largest global therapeutics market [1, 2]. The medicinal Cannabis industry is currently one such focal area, largely because of the occurrence of phytocannabinoids (pCBs). As part of a unique class of phytochemicals that interact with the human endocannabinoid system (ECS), the use of pCBs as therapeutics holds promise in the treatment of numerous chronic diseases [3–8]. However, many countries still abide by strict regulatory laws pertaining to the commercial growth of Cannabis and, consequently research into pharmacologically relevant products has until recently, been relatively protracted. Since the discovery of pCBs, similar compounds (pCB-like) have been reported in several plant species, beyond Cannabis, that are commonly used in TM practices [9]. The growing interest in the use of TMs enabled an opportunity for the discovery and evaluation of new compounds from medicinal and non-medicinal plants for the development of natural therapeutics.

The use of the ‘wild cannabis’ plant, *L. leonurus* (L.) R. Br., endemic to South Africa, is commonly reported in TM to treat numerous ailments including eczema, headaches, hypertension, and chest infections [10, 11]. The leaves (when smoked) have been described to elicit similar effects to Cannabis, including mild psychoactive symptoms, as well as the capacity to alleviate anxiety and induce calming effects [10, 12]. Despite the amount of research available on the leaves, little to no information exists on the medicinal properties of the flowers of this plant. Furthermore, the occurrence of pCBs or pCB-like compounds has never been reported. In this research note, we report on the tentative identification of a pCB-like compound (like adrenoyl-EA) in the flowers of the white flower variety *Leonotis leonurus* var. *albiflora* Benth.

## Main Text

### Materials and methods

#### Plant material

**Fig. 1:**
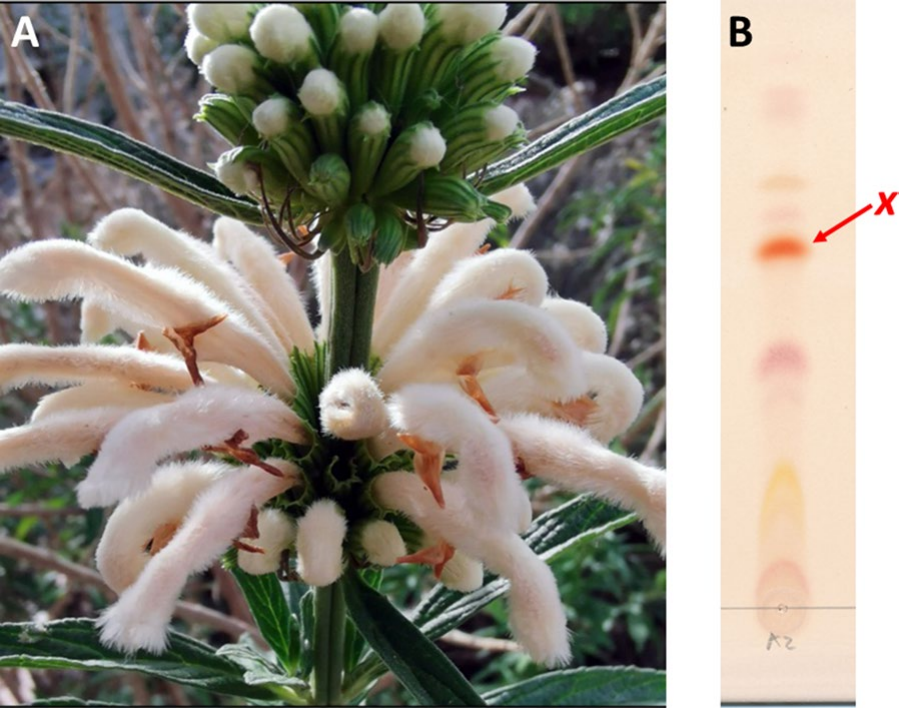
HPTLC phytochemical profile of *Leonotis leonurus* var. *albiflora* Benth. whole flower extract. **a** *L. leonurus* var. *albiflora* Benth. white flower phenotypic appearance (Photo courtesy of Dr B Loedolff) and **b** its corresponding acetonitrile (75%) extract, HPTLC phytochemical profile. Whole flower metabolite extractions were prepared using various extraction solvents and, the acetonitrile (75%) extracts were selected for downstream analyses. The phytochemical profile was developed fully (mobile phase; chloroform:methanol 9:1, stationary phase; silica_60Å_) before reagent visualization (0.1% Fast blue B salt dissolved in 1 mM NaOH) was applied to reveal potential pCB candidates, based solely on an red–orange colour development. Compound *x* (Rf value: 0.55) represents a colorimetric-specific pCB-like candidate compound which was selected for further LC–MS/MS analyses

*L. leonurus* var. *albiflora* Benth. seeds (white flower variety) were obtained from a commercial seed supplier (Seeds for Africa, South Africa) and grown on a residential property at the Bottom Road Sanctuary, Zeekoevlei, Western Cape Province, South Africa (GPS coordinates: −34.057951, 18.499391). Flower samples were harvested at mature stage (Fig. 1, photo courtesy of Dr B Loedolff) and immediately stored at −20 °C.

#### Whole flower metabolite extraction

Flowers were lyophilized (20 h; 100 mTorr, −60 °C) and ground to a fine powder using a pestle and mortar. Metabolites were extracted from flower material (50 mg) as previously described [13, 14], evaporated, and the resin reconstituted in 200 μL ddH_2_O, calibrating the extracts to 0.50 mg/μL.

#### Phytocannabinoid screening and isolation using HPTLC

Phytocannabinoid screening was performed on 10 × 20 cm glass-back plates pre-coated with 0.25 mm silicagel 60 (REF 811213, Macherey–Nagel, Germany). Flower derived-extracts (0.50 mg/μL) were applied to the plates in 2 μL increments and allowed to dry completely before placement into a glass chromatography chamber (Camag, Switzerland), pre-conditioned with the mobile phase, chloroform:methanol (9:1; 30 min). Subsequently, the chromatogram was developed in the dark using fast blue B reagent (0.1% w/v; dissolved in 1 mM NaOH). Based on R_f_ ranges (comparative to the upper R_f_ values representing the less polar range of pCBs from Cannabis, under the same chromatographic conditions [15]) and change in colour [16]), a single, red–orange compound of interest was scraped from the preparative HPTLC plate and resuspended in 50% (v/v) methanol, prior to tandem mass spectrometry (LC–MS/MS) analyses.

#### Tandem mass spectrometry (LC–MS/MS) analyses

LC–MS/MS analyses were performed, as previously described [14], with a Waters Synapt G2 quadrupole time-of-flight mass spectrometer (Waters Corporation, Milford, MA, USA) equipped with a Waters Acquity UPLC. Samples were separated on a Waters UPLC BEH C18 column (2.1 × 100 mm; 1.7 μm) at a flow rate of 0.25 ml/min at 55 °C. Solvent A consisted of 0.1% (v/v) formic acid in water and solvent B was 0.1% (v/v) formic acid in acetonitrile. The mobile phase gradient was initiated at 100% solvent A for 1 min and linearly reduced to 28% solvent A over 22 min. Subsequently, the mobile phase was changed to 40% solvent B over 8 min followed by a 1 min wash step in 100% solvent B before the column was re-equilibrated to the initial conditions for 4 min. Electrospray ionization was applied, and samples were analysed in a negative mode run and a positive mode run. Data was acquired in MS^E^ mode, which consists of a high collision energy scan range of *m/z* 125–1500 and a low collision energy scan from *m/z* 40–1500. The photo diode array detector was set to scan from 220–600 nm. The capillary voltage was set at 3.5 kV and the collision energy either 6 V (low collision energy scan from) or 30–60 V (high collision energy scan), the cone voltage was 15 V, the source temperature 120 °C and the desolvation temperature was 275 °C. The desolvation and cone gas (nitrogen) flows were 650 and 50 L/h, respectively. Sodium formate was used for calibration and leucine encephalin was infused in the background as lock mass for accurate mass determinations, where authentic reference compounds were available. Metabolites were monitored using their deprotonated quasi-molecular ions.

Compounds were tentatively identified using the Metabolomics workbench [17]. The database was searched, using the m/z mass obtained from total ion chromatograms, with parameters set to the negative [M-H]^−^ and positive ion [M + H]^+^ mode, respectively, and a mass tolerance of ± 0.5 m/z. Tentative identification was based on the experimental mass of the specific peaks compared to literature and was carried out in conjunction with independent metabolite repositories, Metabolomics Workbench, PubChem, and METLIN Metabolite and Chemical Entity Database.

### Results and discussion

*L. leonurus* (L.) R. Br. is a drought resistant medicinal shrub endemic to South Africa with a bi-annual flowering season (high-yielding medicinal crop). Anecdotally it is known for eliciting mild psychoactive effects akin to the smoking of Cannabis and, has a long-standing history in traditional healing practices in South Africa. Some studies have alluded to its medicinal activities however, these have largely focused on the leaves and only a few reports have dealt with the flowers [10, 11]. Furthermore, there are no reports that describe the occurrence of medicinal pCBs from the leaves or the flowers. Since pCBs have been proposed as effective TMs for the prevention and/or treatment of chronic diseases [18–20], identification of pCBs (or pCB-like compounds) in plants other than Cannabis *spp*. could present an attractive value proposition to complement the emergent Cannabis industry.

Based on its mild Cannabis-like effect when smoked, we conducted analyses into the flower-derived phytochemicals and the potential presence of pCB-like compounds in *L. leonurus* var. *albiflora* Benth. (Fig. 1a). Since extensive phytochemical profiling of the leaves have never yielded any pCB-like compounds [10, 11], we suspected that such compounds may be present in flowers (akin to Cannabis). Using HPTLC, coupled to fast blue B salt as a selective colorimetric detection method for pCB compounds [14], we extracted flower metabolites with gradient concentrations (50, 75 and 100%) of either methanol or acetonitrile (Additional file 1: Figure S1). The fast blue method detects major neutral cannabinoids such as tetrahydrocannabinol (THC), cannabidiol (CBD), and their cannabinoid acid derivatives THCA and CBDA, among other cannabinoids with high sensitivity and selectivity [15, 16].

**Fig. 2:**
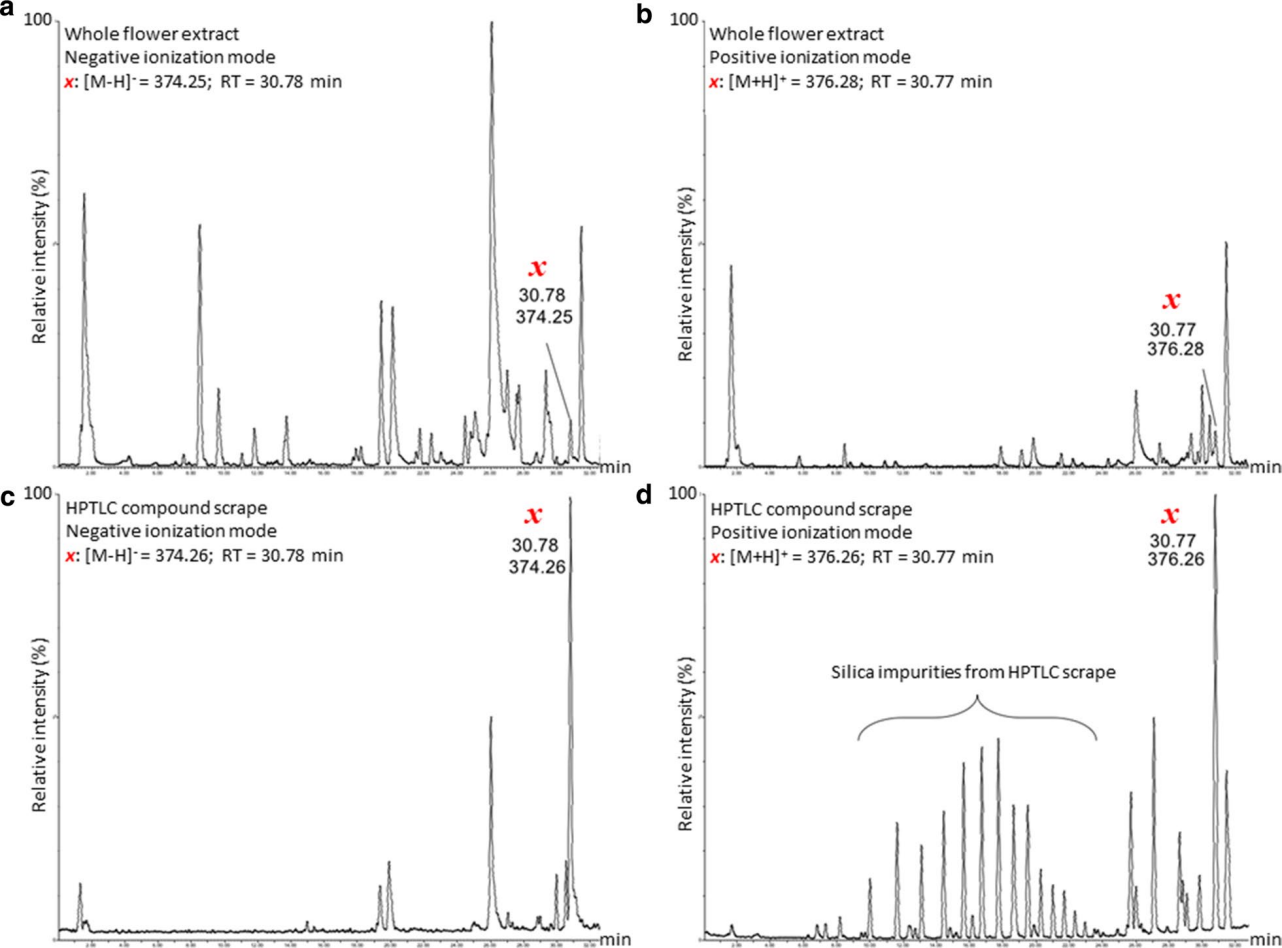
LC–MS/MS spectra of *Leonotis leonurus* var. *albiflora* Benth. whole flower extract, targeting the cannabinoid occurrence in flowers. LC–MS/MS spectra of white *L. leonurus* var. *albiflora* Benth. whole flower extract in negative (**a**) and positive (**b**) ionization modes. Spectra of the preparative HPTLC product, compound *x*, in negative (**c**) and positive (**d**) ionization modes. In accordance with literature, matching retention times and adduct masses for compound ***x***, representing 7,10,13,16-Docosatetraenoylethanolamine, are shown in both ionization modes ([M–H]^−^ = 374.26, RT: 30.78 min; ([M + H]^+^  = 376.26, RT: 30.77 min), where chromatogram axis represent relative mass abundance (%) and retention time (RT, minutes), respectively

A single, potential pCB-like compound (distinct red–orange colour, R_f_ 0.55; Fig. 1b) was isolated, and its identity tentatively determined using LC–MS/MS in both positive and negative ionization modes (Fig. 2a, b). Mass spectra from whole flower extracts and the isolated HPTLC compound were compared (Fig. 2c, d) resulting in equivalent retention times and peak masses in both negative (RT: 30.78 min; [M−H]^−^ = 374.26) and positive (RT: 30.77 min; [M + H]^+^  = 376.26) ionization mode. Further investigation using the international metabolite repositories, Metabolomics Workbench, PubChem, and METLIN Metabolite and Chemical Entity Database, consistently grouped this compound as a fatty amide with tentative identification as 7,10,13,16-Docosatetraenoylethanolamine ([M–H]^−^ = 374.31; [M + H]^+^  = 376.32; neutral m/z = 375.31), also known as adrenoyl-ethanolamide (EA). Adrenoyl-EA is a bioactive endocannabinoid previously thought to be unique to mammals [21, 22]. However, it has recently (and for the first time) been confirmed to occur in methanol extracts of the Mashua plant (*Tropaeolum tuberosum*), extensively used in Andean folk medicine [23]. Our findings tentatively identify the occurrence of a pCB-like compound (predicted to be like adrenoyl-EA) in acetonitrile extracts from the flowers of *L. leonurus* var. *albiflora* Benth.

Adrenoyl-EA is structurally similar to the major endocannabinoid anandamide and functions as an agonist of the CB_1_ and TRPV_1_ receptor proteins, two major receptors of the human ECS [21, 24]. Agonists of these receptors are ideal candidates in a range of therapeutic targets, specifically exhibiting anti-inflammatory, neuroprotective, and anticancer activities, among others [23, 25, 26]. Within the growing market of TMs, we consider it beneficial to identify sustainable, climate resilient plant resources (high yielding medicinal crops) that accumulate pCBs or PCB-like compounds with the potential for therapeutic purposes.

#### A South African perspective on the development of pCB products

It is estimated that the domestic South Africa Cannabis market will reach USD $1.8 billion by 2023 however, this projection is based on export value only, excluding Cannabis-derived pCB products. The value proposition lies within the latter, given the therapeutic potential of pCBs in chronic disease treatment [23–26]. South African agriculture is typified by both large scale mechanized, and smallholder practices and Cannabis cultivation is considered viable. Although the agricultural experience and arable land in South Africa is adequate for the growth and export of Cannabis, the irrigation infrastructure and required daylight might not be sufficient to sustain the production of high quality Cannabis-derived products. One of the major predicted hurdles in establishing a sustainable Cannabis industry is the water-intensive measures required for an efficient pCB yield from Cannabis. If one could exploit a water-efficient medicinal crop capable of producing pCB-like compounds, this would serve as an ideal industry alternative into a realm of “smart-pharming” practices.

As part of a pCB-driven strategy for the development of high value TMs, we suggest that the discovery of pCBs in endemic South African plants could be highly complementary to the Cannabis industry, given that these plants are adapted to the climatic and water-scarce conditions. To our knowledge, we provide the first evidence on the presence of the pCB-like compound, adrenoyl-EA, in the flowers of *L. leonurus* var. *albiflora* Benth. Globally, the production of pCBs from these alternative resources could benefit the future TM market, particularly in countries where agriculture is typified by water scarcity.

## Limitations

This research note serves to highlight the potential existence of pCBs or pCB-like compounds within the whole flower extract of *L. leonurus* var. *albiflora* Benth. HPTLC screening indicated the potential existence of several pCBs, based on the reported selectivity and color index when developed with the fast blue B reagent. From these, we selected one (isolated by scraping the selected area from the HPTLC plate) for downstream LC–MS/MS analyses. The isolated compound was tentatively identified as pCB-like, with adrenoyl-EA predicted as the main candidate on several databases. A major limitation for this study is the absence of an authentic LC–MS/MS reference compound for adrenoyl-EA to (i) calibrate the instrument and accommodate for potential mass shifts/corrections and (ii) compare fragmentation and isotope patterning on our instrument. Results discussed here are tentative based on experimental mass and database searches and, further investigations will be required to unequivocally provide evidence for the exact identity of compounds. These include (i) quantification and confirmation of compound/s with LC–MS/MS using authentic standard compounds and, (ii) determining structural similarities and differences when compared to other plant-, animal- or human-derived adrenoyl-EA, with NMR. However, to support the idea of an alternative and sustainable crop to produce pCB products, it would be beneficial to isolate and identify the other compounds from the HPTLC to uncover the existence of a metabolite pathway capable of producing several different pCB or pCB-like compounds.

## Supplementary information

**Additional file 1: Figure S1.** HPTLC phytochemical profile of *Leonotis leonurus* var. *albiflora* Benth. whole flower extracts, using different solvent concentrations for extraction. Phytochemical extractions were optimized using different concentrations (1, 50%; 2, 75%; 3, 100%, respectively) of either acetonitrile (A) or methanol (M). Mobile phase: chloroform, 10% methanol. Loading volume: 12 µL. pCB-specific compounds were derivatized with fast blue B reagent (dissolved in 0.1%, 1 mM NaOH) under dark conditions. HPTLC plates were captured under visible light following derivatization. A single spot, based on the red–orange colour development (A2; 75% acetonitrile extraction, indicated in the red dotted-line rectangle), was isolated from the HPTLC plate and used for downstream LC–MS/MS analyses.

## Data Availability

Any data, including the full LC–MS/MS datasets, used and/or analysed during the current study are available from the corresponding author (bianke@sun.ac.za) on reasonable request.

## Abbreviations

pCB: Phytocannabinoid
HPTLC: High performance thin layer chromatography
LC–MS/MS: Liquid chromatography linked to tandem mass spectrometry
CM: Conventional medicine
TM: Traditional medicine
THC: Tetrahydrocannabinol
CBD: Cannabidiol
NMR: Nuclear Magnetic Resonance
