# Supplementary figures and images for: Toward the identification of a phytocannabinoid-like compound in the flowers of a South African medicinal plant (Leonotis leonurus)
Source: BMC Res Notes. 2020 Nov 10;13:522. doi: 10.1186/s13104-020-05372-z (PMC7653773; doi:10.1186/s13104-020-05372-z)

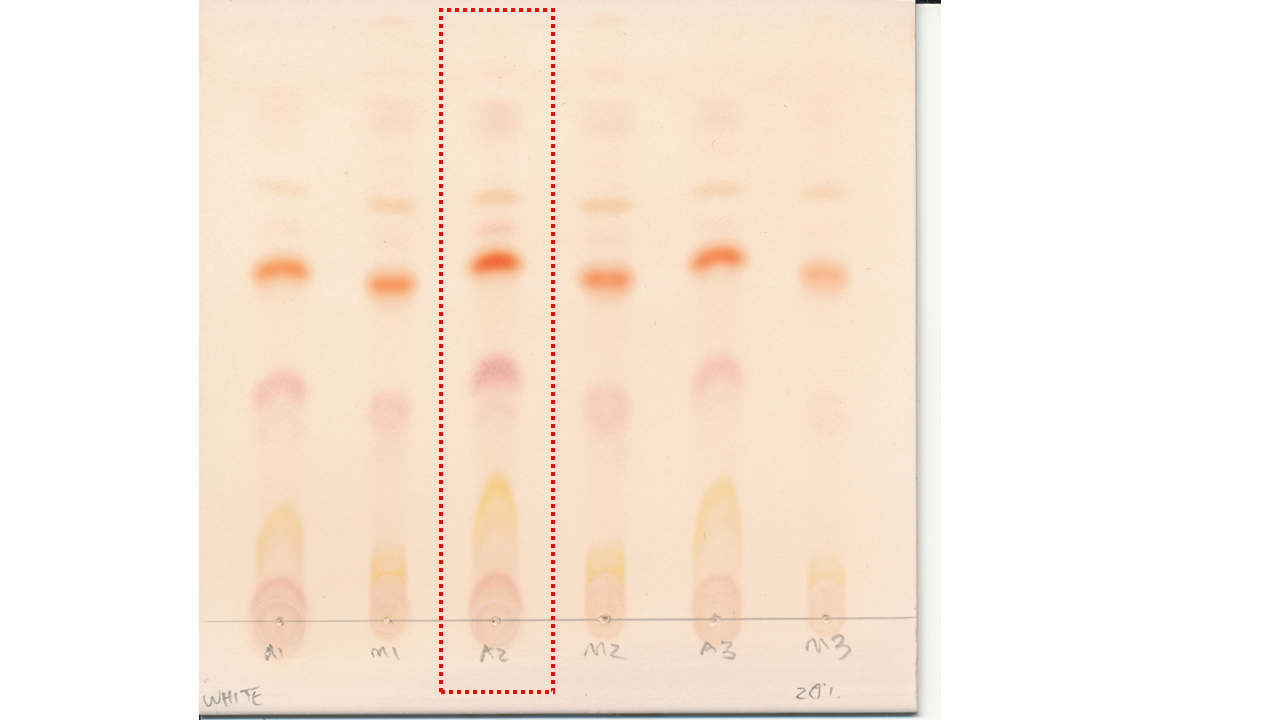

Supplement: Supplementary file 1 — Additional file 1: Figure S1. HPTLC phytochemical profile of Leonotis leonurus var. albiflora Benth. whole flower extracts, using different solvent concentrations for extraction. Phytochemical extractions were optimized using different concentrations (1, 50%; 2, 75%; 3, 100%, respectively) of either acetonitrile (A) or methanol (M). Mobile phase: chloroform, 10% methanol. Loading volume: 12 µL. pCB-specific compounds were derivatized with fast blue B reagent (dissolved in 0.1%, 1 mM NaOH) under dark conditions. HPTLC plates were captured under visible light following derivatization. A single spot, based on the red–orange colour development (A2; 75% acetonitrile extraction, indicated in the red dotted-line rectangle), was isolated from the HPTLC plate and used for downstream LC–MS/MS analyses. [file 13104_2020_5372_MOESM1_ESM.tif]
